# Nitric Oxide-Induced Polycystic Ovaries in The Wistar Rat

**Published:** 2012-06-19

**Authors:** Fatemeh Hassani, Manizheh Karami, Ph.D. 1 1, Mohammad Reza Jalali Nadoushan, Poopak Eftekhari Yazdi

**Affiliations:** 1 Department of Biology, Faculty of Basic Sciences, Shahed University, Tehran, Iran; 2 Department of Pathology, School of Medicine, Shahed University, Tehran, Iran; 3 Department of Embryology, Reproductive Biomedicine Research Center, Royan Institute for Reproductive Biomedicine, ACECR, Tehran, Iran

**Keywords:** PCOS, Nitric oxide, L-Arginine, Naloxone, NADPH-Diaphorase

## Abstract

**Background:**

Nitric oxide (NO) involves in polycystic ovary syndrome (PCOS), a cause of infertility in women during the reproductive age. The PCOS is now categorized as an inflammatory phenomenon. The aim of this study was to evaluate the role of NO, a proinflammatory agent, in this syndrome at histological and biochemical levels.

**Materials and Methods:**

In this experimental study, animals were female Wistar rats (weighing 200-250 g) kept under standard conditions. L-Arginine (50-200 mg/kg), a precursor of NO, was injected intra-peritoneally (i.p.) through a period ranging from 9 to14 days/ once a day. The rats' estrous cycle was studied using Papanicolaou test; those showing phase of Diestrous were grouped into experimental and control groups. The control group solely received saline (1 ml/kg, i.p.) throughout all experiments. To evaluate the inflammatory effect of NO, the rats were treated an anti-inflammatory agent, naloxone hydrochloride (0.4 mg/kg, i.p.), prior to L-arginine. At the end of the treatment period all animals’ ovaries were assessed for histopathological and histochemical investigations. Also, activation of NO synthase (NOS) in the experiments was studied using NADPH-diaphorase technique.

**Results:**

The ovaries of rats treated with L-arginine showed polycystic characteristics in contrast to those collected from control or naloxone pretreated groups, based on image analysis. A difference in enzyme activation was also shown in the sections that belonged to the groups that received L-arginine when compared with the pre-naloxone and control groups.

**Conclusion:**

Based on these results, we believe that NO may play a major role in the pathophysiology of PCOS.

## Introduction

Polycystic ovarian syndrome (PCOS) is introduced as common endocrine disorder that affects up to 10% of women during their reproductive ages (1). This disoreder is manifested by heterogeneous clinical features (1-4) as it characterized by hirsutism, irregular menstrual cycles and infertility (5). In anovulatory women suffering from PCOS the prominent ovarian signs are listed as the follicular maturation arrest (6) and insulin resistance (5). The later phenomenonhas been related with impaired production/release of endothelium-derived nitric oxide (NO) and increased levels of endothelin-1 known as main markers of vascular disease (5).

NO is indicated as an important paracrine messenger that participates in several physiological and pathophysiological events in the endocrine organs (7). This molecule is a free-radical produced from the oxidation of terminal guanidino nitrogen of arginine by the action of nitric oxide synthase enzyme (NOS) (7). The molecule is well documented as a local inflammatory generator and included with the factors introduced as those responsible for the ovulatory processes and the PCO syndrome as well (8). Naloxone, a narcotic drug, is used as a novel antiinflammatory agent (9).

This study evaluates the influence of L-arginine, a precursor of NO, in PCOS-induction in female Wistar rats. NADPH-diaphorase provides valuable biochemical data representing the participation of NO in this phenomenon. The marker is classified as the best indicator of NOS enzyme. We have also examined the metabolic status in L-arginine-exposed rats and evaluated the effect of naloxone in L-argininetreated rats.

## Materials and Methods

### Animals

In this experimental study, the animals were eight-week-old female Wistar rats (weight: 200-250 g) purchased from Pasteur Institute of Iran (Tehran, Iran). Animals were retained under standard conditions in accordance to the Guide for the Care and Use of Laboratory Animals (1). Animals were maintained in a standard temperature (21 ± 3˚C) and 12 hours light/ dark cycle with food and water ad libitum. All experiments were approved by the local Ethical Committee at Shahed University.

### Drugs

L-arginine, nitroblue tetrazolium (NBTS), and β-nicotinamide adenine dinucleotide phosphate (β-NADPH) were purchased from Sigma Chemical Co. (St. Louis, MO, USA). Naloxone hydrochloride was provided by the Tolid Daru Co., Tehran, Iran. Ketamine and xylazine were obtained from Veterinary Organization, Tehran, Iran.

### Papanicolaou (PAP) stain

Female rats have a 4-5 day cycle, including proestrous (12 hours follicular growth and peak estrogen phase), estrous (12 hours ovulation phase), metestrous (12 hours corpus luteum secrets progesterone phase), and diestrous (48 hours corpus luteum regression phase). Without exposing with male rats, the female rats are always in the diestrous phase; their phases will progress only after mating with a male rat (10, 11). With regards to the above-mentioned explanation, we chose virgin rats in our study. Because vaginal changes may appear through the experiments, the animals’ vaginal smears were also examined (1) during the entire experiment by the Papanicolaou (PAP) stain. In this procedure the smears obtained by vaginal washing between 11:00 am and 12:00 pm were stained using a PAP stain for humans, as developed in previous studies (12, 13). Three types of cells were recognized in the PAP stained smears: i. round and nucleated epithelial cells;ii. irregular cornified cells; iii. little round cells (leukocytes). The cell proportion in the smear was used to determine the estrous cycle phase as previously been proposed (12, 13).

### Drug administrations

Female Wistar rats (200-250 g) at eight weeks of age were randomly divided into the L-arginine dose groups (50-200 mg/kg; n=10 in each) and a group that was pre-injected with naloxone (0.4 mg/kg; n=10) prior to L-arginine administration. The drug, L-arginine, was injected intra-peritoneally (i.p.) once daily during a period that ranged from 9 to 14 days. Naloxone was injected once per day 30 minutes prior to L-arginine administration. The control group received saline (1 ml/kg i.p.) instead of the drugs.

### Surgery procedure for removal of ovaries

The treatment groups were anesthetized by diethyl ether in a special device. Then a 2 cm midline incision in the lower abdomen area was performed. The ovaries were carefully examined biometrically and dissected out, then collected in a fixative for further histological and biochemical analysis.

### Histological analysis

For histological investigations, the ovaries were fixed in a 10% formalin solution and processed with a tissue processor through paraffin embedding. Serial sections (3-5 μm) were prepared with a rotary microtome. The slides were then stained using the hematoxylin and eosin (H&E) staining method (6) and cleared with xylene. After mounting, the slides were evaluated with a light videophotomicroscope (Olympus, USA and a videophotobinocular at the desired magnification.

### Hormone assays

#### Blood sampling for serum parameters

At the end of the experiment, the rats were anesthetized by i.p. injections of 100 mg/kg ketamine hydrochloride and 20 mg/kg xylazine. Heart blood samples were obtained between 8:00-10:00 am after an overnight fast to assess sugar, lipid, and hormonal profiles. All samples were kept at room temperature for at least 30 minutes to facilitate the clotting of the samples. The samples were then centrifuged at 3000- 5000 g for 15 minutes. The sera were stored at −20˚C for future assessments. The level of sera agents [high-density lipoprotein (HDL), low-density lipoprotein (LDL), glucose, cholesterol, and triglycerides] were measured based on competition binding using the enzyme-linked immunosorbent assay (ELISA) and read with an ELISA reader. Other tests were performed by using a Cobas Mira plus CC Chemistry Analyzer as previously mentioned (1, 5, 6).

#### NADPH-diaphorase reactivity

The activation of nitric oxide synthase (NOS) enzyme in the ovarian tissue was shown using the NADPH-diaphorase at the histochemical level. Rats were anesthetized 24 hours after the last injection, and then the ovaries were excised and trimmed from periovarian fat and bursae. The samples were then immersed in phosphate buffered saline (PBS, pH=7.2) overnight at 4˚C, followed by cryoprotection in 30% sucrose. Then specimens were sectioned with cryostat microtome at -15˚C (9-10 μm frozen sections) and mounted on poly-L-lysine-coated slides (7, 14). The slides were rinsed with buffer and then stained using the nicotinamide adenine dinucleotide phosphate (NADPH)-diaphorase technique to visualize NOS activity. Briefly, the prepared slides were incubated with shaking state in a 0.3% Triton-X 100 in phosphate buffer for 1 to 2 minute(s). The staining was then performed by incubating the slides in a solution containing equal parts of nitro-blue tetrazolium (NBT, 0.2 mg/ml in buffer) and NADPH (1 mg/ml in buffer) for about 16 hours at 37˚C. Upon reduction by NADPH-diaphorase, NBT yields a blue formazan that is visible by light microscopy. Control specimens were assessed using the same procedure except that the specimens were placed in an incubation bath devoid of the marker. No reaction to NADPH was observed in the control samples. The tissue samples were then dehydrated in ascending series of ethanol, cleared in xylene alcohol and xylene, and mounted in Entellan (Merck co., Germany) (7, 15).

### Image analysis

The tissue slides were observed under the videophotolightmicrocope (Olympus, USA and the provided images were then assessed in 100-μm^2^ units of the photorecords at ×4 or more magnification using the Image Tool program (UTHSCSA, version 2.03), the free image processing and analysis program for Microsoft Windows to provide quantification for an area of 100 μm^2^.

### Statistical analysis

Data were analyzed by the Kolmogorov–Smirnov (K-S) test for showing the equality to analysis by variance (ANOVA) using SPSS software (version 13.0; SPSS Inc., Chicago, IL). A Tukey-Kramer or LSD post hoc test was used to calculate the difference between the groups after ANOVA. Statistical significance was considered p<0.05. All data were expressed as mean ± SEM. In case of the NADPHdiaphorase technique, the intensity analysis was assessed at 100 μm^2^ using the Image Tool program (UTHSCSA, version 2.03), after calibrating for a 100 μm^2^ area.

## Results

### Papanicolaou (PAP) stain

**Fig 1:**
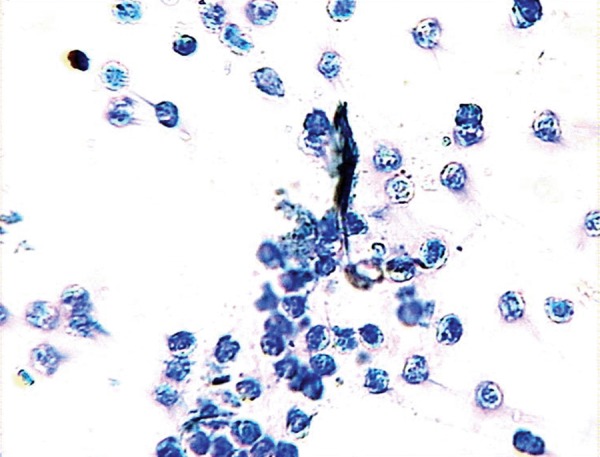
Result of Papanicolaou (PAP) stain of female virgin rat vaginal smears in the diestrous phase. The abundance of leukocytes in the smear samples indicate that the cycle phase is diestrous.

The estrous cycle phase of female virgin rats was determined as diestrous after using the PAP stain (Fig 1). Round, nucleated (epithelial) cells were the most abundant cell type observed in the smears.

### Histology

**Fig 2:**
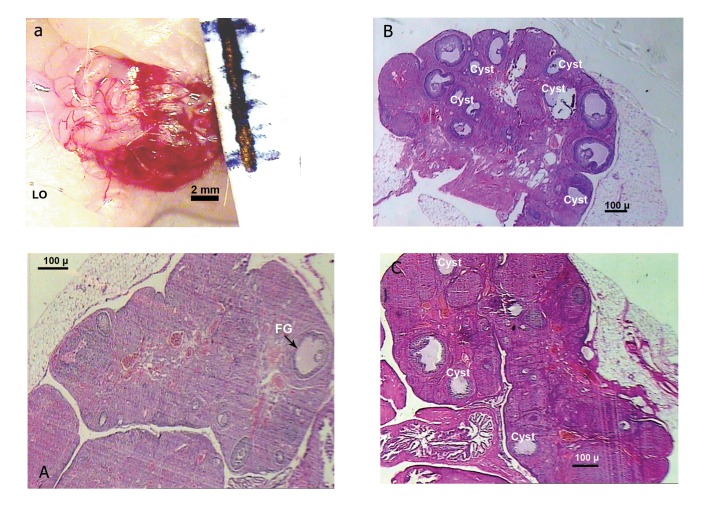
Pictures of ovaries from control (a, A), solely L-argininetreated (B), and naloxone pre-treated (C) rats. Bars beside the samples show mm values. The figures are shown under magnification [a: ×1.6 objective of an (inverted) photomicroscope; A, B, C: Olympus photomicroscope at × 4].

The rats’ ovaries from the L-arginine dose groups (50-200 mg/kg) exhibited small follicles in the early development phase in addition to the follicles that showed evidence of either atresia, large cysts with thickened granulosa cell layer, or large cystic follicles with scant granulosa cells (Fig 2B). The samples of those pre-administered with naloxone (0.4 mg/kg; Fig 2C) showed a reduction in the incidence of cyst formation. The control group exhibited a follicular appearance depending on the phase of the cycle; in diestrous, only secondary follicles and fresh corpora lutea were seen. Atretic follicles were also observed in the phases of the control group. The corpus luteum was absent in all cases (Fig 2A).

### NADPH-diaphorase reactivity

**Fig 3:**
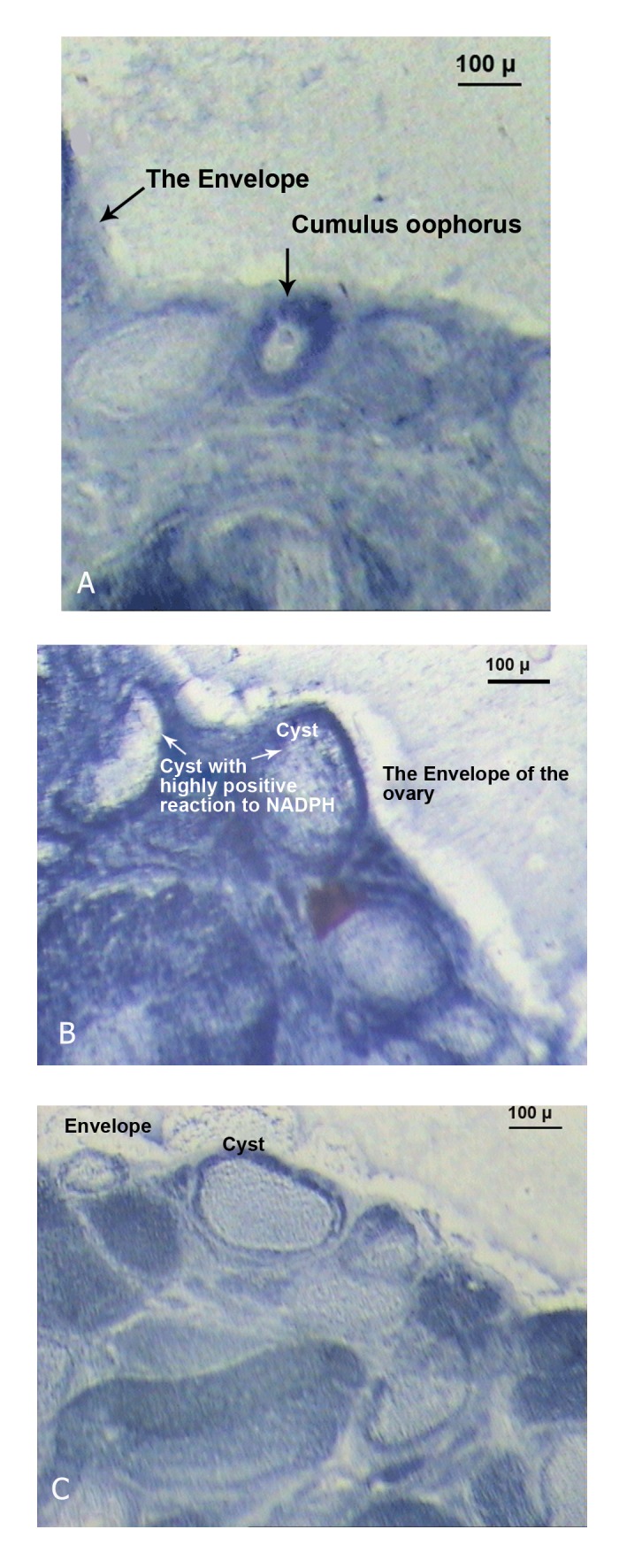
Histochemical NADPH-diaphorase evaluated in control (A) and treated rats; single L-arginine (B) or L-arginine + naloxone (C) as detailed in materials and methods.

Positive NADPH-diaphorase reactivity was observed in the stroma and in the theca cell layer in the ovaries of L-arginine treated rats (Fig 3A). The reactivity was also observed in the membrane granulosa cell area of the follicles in the samples. In luteinized ovaries, weaker diaphorase reactivity was observed in patches at the periphery of the corpora lutea. The response to NADPH-diaphorase showed attenuation in naloxone pre-injected samples (Fig 3B). In a comparison analysis completed by Image tool, no significant reaction was observed in the control specimen (Fig 3C). It should be noted that the areas of interest contained NO-generating enzyme activity, and that the activity of NOS was in agreement with the time of rupture and cystic formation.

### Serum agents levels

**Fig 4:**
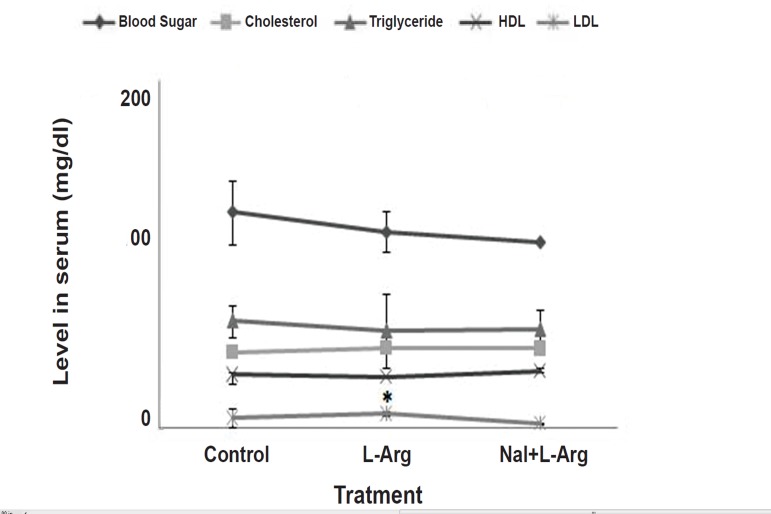
Curves indicate the levels of metabolic agents in serum (mg/dL) both in the control and experimental groups (n=10). The experimental rats received L-arginine (50 mg/ kg, i.p., 14 days) or L-arginine (50 mg/kg, i.p., 14 days) plus naloxone (0.4 mg/kg, i.p., 14 days). Naloxone was injected 30 minutes prior to L-arginine. Values are mean ± SEM. * p<0.05 vs. control (Tukey or LSD test).

Levels of serum parameters are shown in Fig. 4. As the data denotes, L-arginine treatment induced a significant change in the level of LDL in contrast with the control. The naloxone group showed no significant difference to the control, meaning that the agent integrated the deviation level (Fig 4).

## Discussion

NO is categorized as an important intra-ovarian mediator (16-19) that affects the ovulatory process and regulates the functions of the corpus luteum. The present study has lent further support for a functional role of NO in the ovarian functions. Activation of the NOS enzyme was demonstrated using NADPH-diaphorase. The present findings showed the activity of this enzyme both in the stromal and thecal layers in ovaries treated with L-arginine.

This study provided more evidence for follicular atresia, as well as the production of large cysts due to NO treatment in Wistar rats, which agreed with a previous finding. Despite the vascular effect of NO, which forms the highly reactive cytotoxic peroxynitrite (20), it may mediate the cellular cytotoxicity of ovaries by the PCOS phenotype effect and via cytotoxic peroxynitrite. NO production and formation of the free radicals may have some importance in the ovulatory process. This fact may facilitate theca tissue events. The tunica albuginea of the follicle wall may be one of the symptom’s signs as been noted previously (21). In the present study these areas have been found to contain NO-generating enzyme activity and the NOS activity agreed with the time of rupture and cystic formation.

Except for the level of serum LDL (Fig 4), no significant change in other levels, such as glucose or blood sugar, was found. Na´cul et al. (5) showed a negative correlation between NO and markers of glucose homeostasis in PCOS patients. The NO levels appeared to be regulated by endogenous estrogens because a higher level was described in the follicular phase than in the luteal phase of the menstrual cycle (22). These data possibly suggest that the estrogen levels in the diestrous phase may interfere with NO secretion, which is presumably related to the presence of PCOS.

Though no significant change in sera levels was found, the administration of naloxone prior to L-arginine led to a decrease in the level of serum LDL (Fig 4) and attenuated NO-producing activity (Fig 3). These findings possibly demonstrate that the NO molecule, as a proinflammatory element, may induce PCOS by activating inflammatory factors in ovaries, and the anti-inflammatory agent naloxone affects the endocrine and metabolic measures relevant to PCOS. Naltrexone administration has been shown to result in reduced food intake in android obesity (23). Metabolic abnormalities such as hyperinsulinemia, insulin-resistance, and obesity, are listed as common features of PCOS. A link between opioids and PCOS-related insulin response to glucose load has also been previously suggested (24, 25).

## Conclusion

The findings show a correlation between central opioid tone and body weight. The regulation of metabolic agents by naloxone in this study beyond the effect of the drug on PCOS signs remains elusive.
